# Preclinical Evaluation of the Effect of Liquid Platelet–Rich Fibrin and a Volume‐Stable Collagen Matrix on Periodontal Regeneration

**DOI:** 10.1111/jcpe.70065

**Published:** 2025-11-23

**Authors:** Jean‐Claude Imber, Andrea Roccuzzo, Nikola Saulacic, Maria Permuy Mendaña, Sıla Cagrı Isler, Dieter D. Bosshardt, Anton Sculean, Alexandra Stähli

**Affiliations:** ^1^ Department of Periodontology, School of Dental Medicine University of Bern Bern Switzerland; ^2^ Shanghai Perio‐Implant Innovation Center, Institute of Integrated Oral, Craniofacial and Sensory Research, Shanghai Ninth People's Hospital Shanghai Jiao Tong University School of Medicine Shanghai China; ^3^ College of Stomatology, Shanghai Jiao Tong University, National Center of Stomatology, National Clinical Research Center for Oral Diseases Shanghai Key Laboratory of Stomatology Shanghai China; ^4^ Department of Cranio‐Maxillofacial Surgery, Inselspital, Bern University Hospital University of Bern Bern Switzerland; ^5^ Department of Veterinary Clinical Sciences University of Santiago de Compostela Lugo Spain; ^6^ Department of Periodontology, Faculty of Dentistry Gazi University Ankara Turkey

**Keywords:** animal model, histology, micro‐CT, periodontal regeneration, platelet‐rich fibrin

## Abstract

**Aim:**

To evaluate the regenerative potential of liquid platelet–rich fibrin (PRF), with and without a volume‐stable collagen matrix (VCMX), in periodontal intrabony defects using histological, histometric and micro‐CT analyses.

**Materials and Methods:**

One‐wall intrabony defects were surgically created in six beagle dogs and treated with either VCMX plus liquid PRF (TG1), liquid PRF alone (TG2), VCMX alone (CG1) or left empty (CG2). After 12 weeks, samples were analysed by micro‐CT, histology and histometry.

**Results:**

Healing was uneventful in all animals. TG1 showed the greatest cementum (4.08 ± 0.88 mm) and bone formation (3.42 ± 0.70 mm), followed by TG2 (2.92 ± 1.13 mm cementum, 3.19 ± 0.78 mm bone) and CG1 (2.07 ± 1.06 mm cementum, 3.29 ± 0.77 mm bone). CG2 exhibited the poorest outcomes (0.84 ± 0.74 mm cementum, 2.58 ± 0.38 mm bone). Micro‐CT confirmed higher bone volume in TG1 and TG2 compared to CG2; however, the difference was not significant. All treatment groups showed significantly greater cementum and bone formation compared to CG2.

**Conclusions:**

Within its limitations, the present study indicates that (i) liquid PRF, alone or combined with a volume‐stable collagen matrix, enhances periodontal regeneration more than open flap debridement alone, and (ii) the combination tends to show the most favourable outcomes.

## 1 Introduction

Over recent decades, regenerative procedures have shown clear benefits in intrabony and furcation defects compared with access flap surgery alone (Avila‐Ortiz et al. 2015; Castro et al. 2017; Cortellini and Tonetti 2015; Kao et al. 2015; Miron et al. 2016; Miron, Shirakata, et al. 2025; Sanz et al. 2015; Sculean et al. 2015; Sculean et al. 2008). However, outcomes remain less predictable in non‐contained defects, where space maintenance and wound stability are critical for healing (Sculean et al. 2015; Susin et al. 2015).

A volume‐stable collagen matrix (VCMX) was developed to address these challenges through high porosity and mild cross‐linking, supporting clot stabilisation and volume preservation (Asparuhova et al. 2021; Imber et al. 2021; Mathes et al. 2010). Preclinical and clinical studies confirmed its biocompatibility, tissue integration and angiogenic potential (Caballe‐Serrano et al. 2019; Ferrantino et al. 2016; Thoma et al. 2020; Thoma et al. 2010; Thoma et al. 2016; Zeltner et al. 2017). In two‐wall intrabony defects, VCMX combined with open flap debridement led to significantly greater cementum and bone formation than debridement alone (Imber et al. 2021). Whether similar effects occur in more demanding one‐wall defects remains unknown.

Collagen matrices may also act as carriers for autologous platelet concentrates (APCs) such as platelet‐rich fibrin (PRF) (Al‐Maawi et al. 2019; Quirynen, Sculean, et al. 2025). PRF, prepared without anticoagulants, contains platelets and leukocytes that release growth factors, promote fibroblast and endothelial activity and modulate inflammation (Barootchi et al. 2025; Bennardo et al. 2025; Blanco, Caramês, and Quirynen 2025; Ghanaati et al. 2018; Miron, Moraschini, et al. 2025b; Miron et al. 2017; Perussolo et al. 2025; Quirynen, Blanco, et al. 2025; Quirynen et al. 2023; Siawasch et al. 2025; Valentini et al. 2025). Low‐speed centrifugation protocols have further enhanced PRF's growth factor release, angiogenic response and leukocyte enrichment (Blanco, García, et al. 2025; Choukroun and Ghanaati 2018; Dohan Ehrenfest et al. 2018; El Bagdadi et al. 2019; Fujioka‐Kobayashi et al. 2017; Gruber 2025; Kubesch et al. 2019; Wend et al. 2017). Although clinical studies show improved attachment gain and bone fill with PRF, histological data on its regenerative potential remain limited (Ghanaati et al. 2018; Miron, Moraschini, et al. 2025a). As PRF alone lacks space‐maintaining capacity, combining it with a stable scaffold like VCMX may offer synergistic biological and mechanical benefits.

Therefore, the primary aim of this study was to histologically evaluate the regenerative potential of VCMX as a carrier for liquid PRF in one‐wall intrabony defects in dogs. The secondary aim was to assess the regenerative effect of liquid PRF alone in the same model.

## 2 Materials and Methods

### 2.1 Animals

Six healthy female beagle dogs (20–22 months, 12–15 kg; Isoquimen, Barcelona, Spain) were housed under controlled laboratory conditions (15°C–21°C, > 30% humidity) at the Rof Codina Foundation (Cebiovet, Lugo, Spain) with ad libitum access to water and a standard diet. Environmental enrichment and daily veterinary supervision were provided. The study complied with Directive 2010/63/EU, was approved by the Rof Codina Ethics Committee (03/20/LU‐001) and adhered to ARRIVE guidelines (Percie du Sert et al. 2020).

### 2.2 Study Design and Sample Size

This split‐mouth, randomised controlled study included two test and two control groups. All sites received open flap debridement (OFD), with treatment allocation randomised using a computer‐generated randomisation sequence (via www.randomization.com).

− Test group 1 (TG1): VCMX (Geistlich Fibro‐Gide, Geistlich Pharma AG, Wolhusen, Switzerland) with liquid PRF.
− Test group 2 (TG2): Liquid PRF.
− Control group 1 (CG1): VCMX, positive control.
− Control group 2 (CG2): Empty, negative control.

Sample size estimation was based on a previous animal study showing significant differences in periodontal regeneration among one‐, two‐ and three‐wall defects (Kim et al. 2004). Reported effect sizes were large (Cohen's *f* = 1.02 for junctional epithelium, 1.15 for cementum, 0.64 for bone). Assuming α = 0.05% and 80% power, even moderate effects (*f* = 0.25) would be detectable. Six animals with eight intrabony defects each (total 48 sites) were included, with two defects per group per animal (12 sites per group). The animal served as the experimental unit (*n* = 6); accounting for within‐animal correlation, the effective sample size per group was estimated at 6–8. Given the large reference effects, the design provides > 90%–99% power to detect group differences. All analyses were performed under blinded conditions.

### 2.3 PRF Preparation

Venous blood was collected from each animal using two sterile 10‐mL S‐PRF tubes (Process for PRF, Nice, France) and centrifuged immediately (PRF DUO Quattro, fixed‐angle rotor, 110 mm radius) at 600 rpm (44*g*) for 8 min following the low‐speed centrifugation concept. The upper 1 mL of the orange‐yellow liquid PRF layer above the red blood cell fraction was collected with a syringe for use.

### 2.4 Surgical Procedure

**FIGURE 1:**
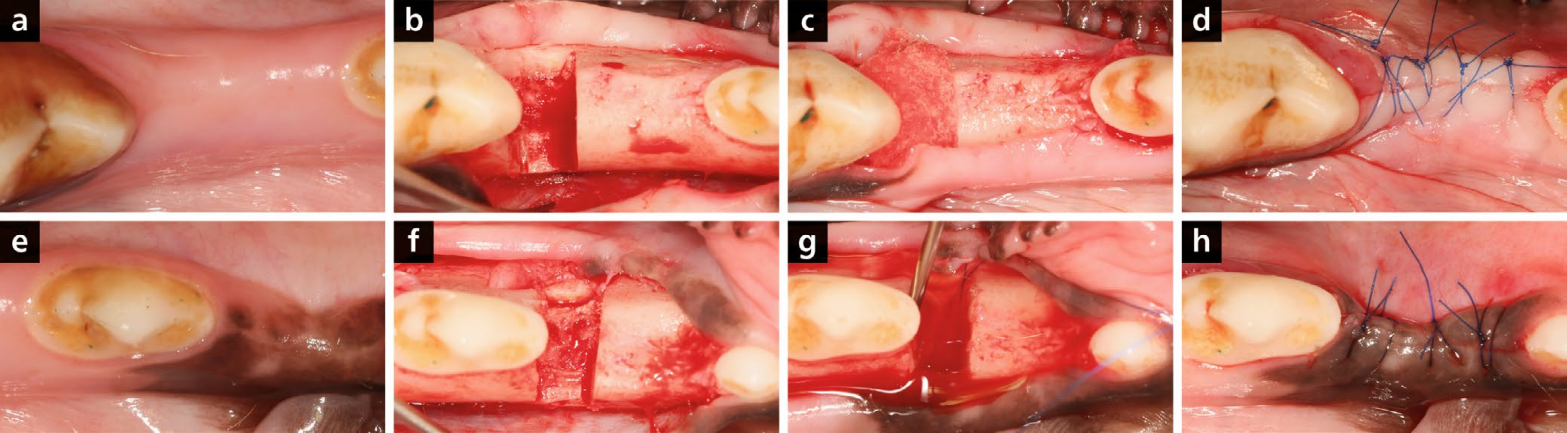
Clinical images illustrating the procedure in TG1 (VCMX + liquid PRF, a, b, c, d) and TG2 (liquid PRF, e, f, g, h): After defect creation (b, f); after application of VCMX socked with liquid PRF (c) or liquid PRF alone (g); flap closure (d, h).

In phase 1, animals were pre‐anaesthetised with medetomidine (10 μg/kg IM) and morphine (0.3 mg/kg IM), induced with propofol (2 mg/kg IV), and maintained with 1%–2% isoflurane in O₂. Local lidocaine/adrenaline was used to minimise pain and bleeding. The maxillary first and fourth and mandibular second and fourth premolars were extracted, followed by 12 weeks of healing (Figure 1a,e).

In phase 2, animals were anaesthetised as before. An experienced periodontist (J.‐C.I.) performed all surgeries. Mucoperiosteal flaps were raised, and coronal reference notches (Notch A) were made at the alveolar crest. Standardised one‐wall intrabony defects (~5 × 5 mm) were created mesially to the second and distally to the third premolars (maxilla) and mesially to the second premolars and first molars (mandible) (Figure 1b,f). Root surfaces were planed, and apical notches (Notch B) marked the defect depth. Liquid PRF was prepared, and defects were treated as follows: VCMX + PRF (TG1, Figure 1c), PRF alone (TG2, Figure 1g), VCMX alone (CG1) or left empty (CG2).

Flaps were sutured (Ethilon 6–0; Figure 1d,h). Postoperative care included morphine (0.3 mg/kg IM every 6 h for 24 h), meloxicam (0.1 mg/kg PO for 3 days), cefazoline (22 mg/kg SC × 2) and cefovecin (8 mg/kg SC × 1). Animals were monitored daily. Oral disinfection with 0.12% chlorhexidine was done three times weekly for 2 weeks, followed by plaque control with 0.2% chlorhexidine gel and a soft pellet diet. Sutures were removed after 2 weeks. Euthanasia was performed 12 weeks postoperatively via medetomidine sedation and sodium pentobarbital overdose (100 mg/kg IV).

### 2.5 Histological Procedures and Descriptive Histology

After euthanasia, mandibles and maxillae were removed, and bone blocks containing the biomaterials and surrounding tissues were fixed in 10% formalin. Histological processing followed Imber et al. (2021). From 12 defects per group, 9 were randomly selected, dehydrated in graded ethanol and embedded in methyl methacrylate (MMA). After polymerisation, specimens were sectioned mesio‐distally (Varicut VC‐50), mounted on Plexiglas and ground to 100–150 μm (Knuth‐Rotor‐3). Sections were stained with toluidine blue/McNeal and basic fuchsin.

The remaining three defects per group were decalcified in 10% EDTA, sectioned at 8 μm and stained with haematoxylin and eosin. Paraffin sections were preserved for immunohistochemistry. Images were captured with a digital camera (AxioCam MRc) on a light microscope (Axio Imager M2, Zeiss). All MMA‐ and paraffin‐embedded specimens were used for descriptive and histometric analysis.

### 2.6 Histometric Analysis

Sections showing the most central part of the defect with clearly visible apical and coronal notches were selected for histometric analysis. Histometry on MMA and paraffin sections followed a validated protocol (Imber et al. 2021). Regions of interest were digitised with a light microscope (Axio Imager M2, Zeiss), and histometric landmarks were identified. Because cementum formation was sometimes discontinuous, both continuous and highest cementum points were assessed (Imber et al. 2021). Two independent examiners (J.‐C.I. and S.C.I.) performed all measurements in Zen Pro (Zeiss) along the root axis from the cemento‐enamel junction to the apex.

1. Height of the defect (in mm, apical end of notch A to apical end of notch B).
2. Highest point of new cementum (in mm and percentage, between notch A and notch B).
3. Height of new continuous cementum (in mm and percentage, between notch A and notch B).
4. Height of new bone (in mm and percentage, between apical end of notch B and most coronal point of newly formed bone).
5. Height of the junctional epithelium (JE) including gingival sulcus (in mm).
6. Height of the connective tissue adhesion (in mm, apical end of the JE to most coronal end of the newly formed cementum).

Percentages of new cementum (highest point and continuous) and new bone were calculated relative to defect height.

### 2.7 Micro‐CT Analysis

Samples were scanned using a high‐resolution micro‐CT system (micro‐CT 40, Scanco Medical AG, Brüttisellen, Switzerland) with the following parameters: 70 kV, 114 μA, 1000 projections/180° and a 2048 × 2048 matrix. A 20‐mm holder provided 10 μm voxel size with 3 s integration time. Images were reconstructed in NRecon (Bruker microCT NV) using Feldkamp's algorithm (smoothing = 2, beam hardening = 20, ring artefact = 4) with identical parameters and misalignment correction. Each defect was reconstructed separately. Orientation was standardised in DataViewer by aligning mesial and distal root centres; coronal and transaxial views were exported. The defect volume of interest (VOI) was defined between the apical and coronal notches. Bone volume fraction (BV/TV) was calculated within a standardised VOI of 150 slices (2700 μm) from the apical notch. Mean defect height and new bone height along the root were measured, with bone height expressed as a percentage of defect height.

### 2.8 Statistical Analysis

Data were analysed using Prism v7 (GraphPad Software, La Jolla, CA, USA). Normality was tested with the Shapiro–Wilk test and verified using Q–Q plots. As data were non‐normally‐distributed, group differences were assessed with the Friedman test, followed by post hoc Dunn's correction (six degrees of freedom). For each dog, the two defects per group were averaged to represent one biological unit. Statistical significance was set at *p* < 0.05.

## 3 Results

### 3.1 Clinical Findings

Postoperatively, all animals showed mild swelling, which resolved within 3–4 days. Healing was uneventful, with no wound dehiscence or other complications observed.

### 3.2 Descriptive Histology

**FIGURE 2:**
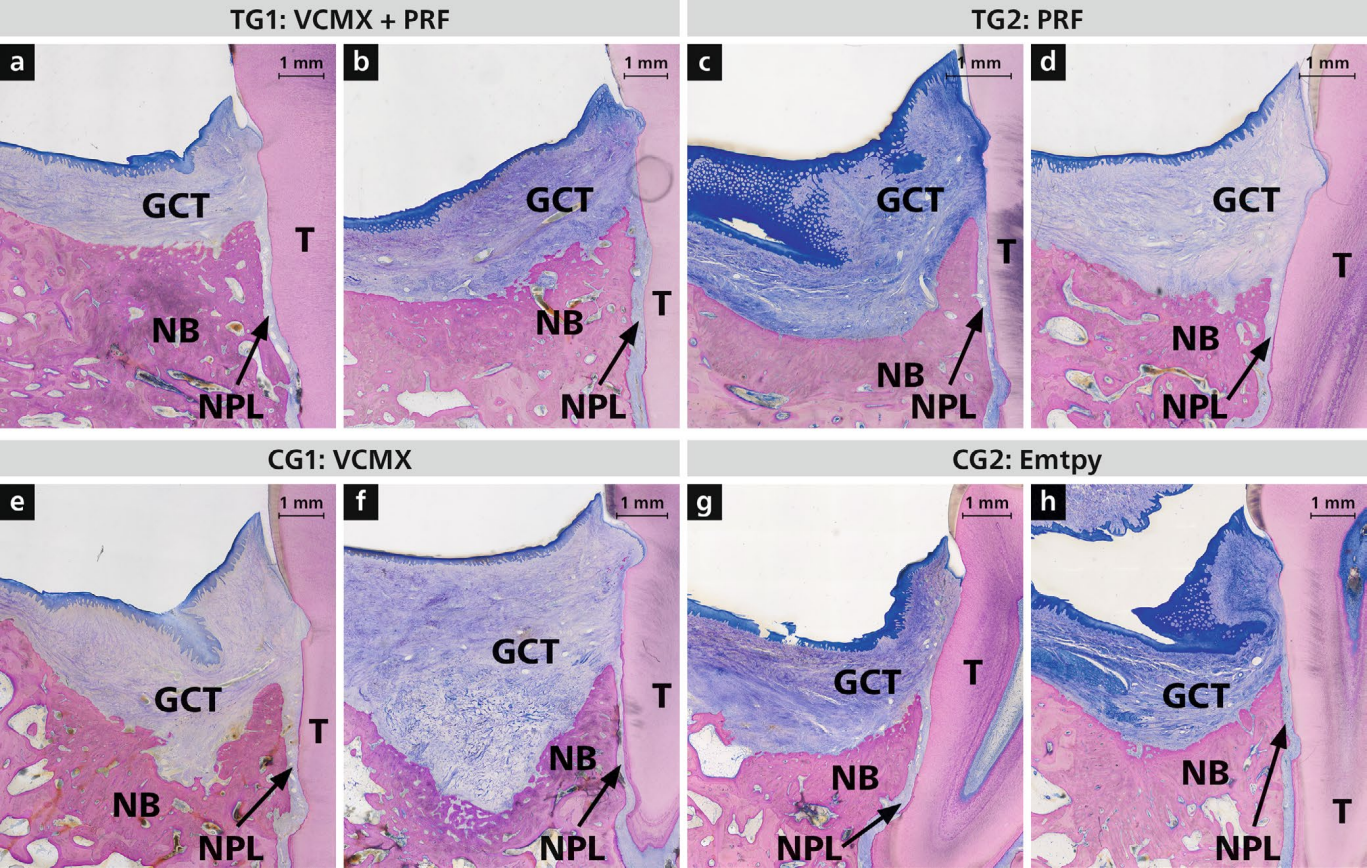
Two representative overviews of histological sections per group: TG1 (VCMX + liquid PRF, a, b), TG2 (liquid PRF, c, d), CG1 (VCMX, e, f) and CG2 (empty, g, h). GCT, gingival connective tissue; NB, new bone; NPL, new periodontal ligament; T, tooth. Staining: toluidine blue/McNeal + basic fuchsin.

Forty‐eight defects were available for descriptive histological analysis—36 embedded in MMA and 12 in paraffin. All defects showed varying degrees of newly formed cementum, alveolar bone and periodontal ligament (PDL) (Figure 2). Two cementum patterns were observed: continuous cementum extending coronally from the apical defect margin, and discontinuous cementum with isolated coronal deposits. Notably, continuous cementum formation was seen in 7 of 12 TG1 defects, but in none of the TG2, CG1 or CG2 samples.

**FIGURE 3:**
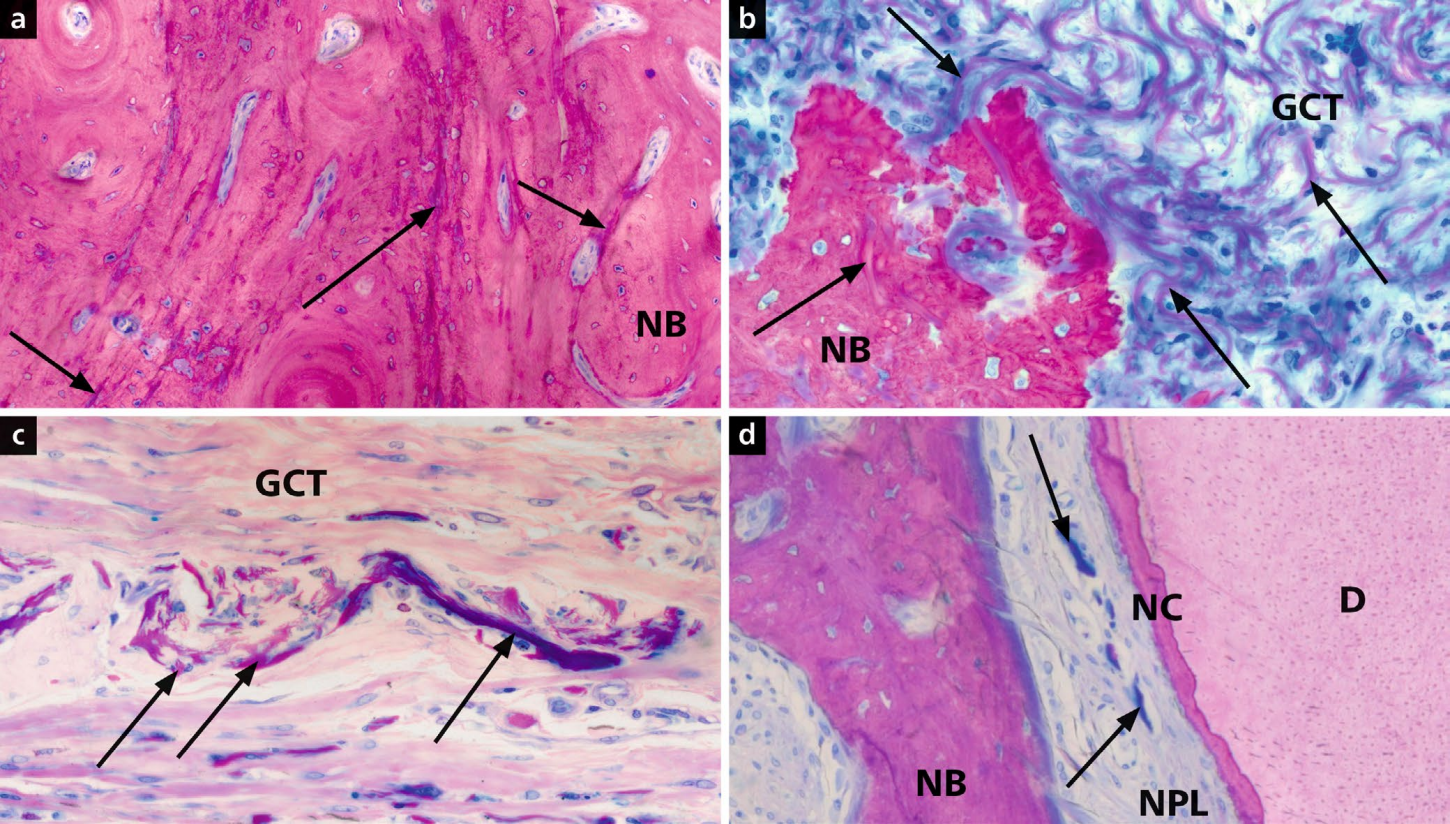
Micrographs illustrating integration of collagen matrix residues (VCMX, arrows) into new bone (NB, a, b), soft connective tissue (CT, c) and new periodontal ligament (NPL, d) in the coronal defect region. D, dentine; NC, new cementum. Staining: toluidine blue/McNeal + basic fuchsin.

Residual VCMX material was present in both TG1 and CG1 (Figure 3), with more advanced degradation in TG1, suggesting enhanced matrix remodelling due to PRF. In mid‐defect areas, VCMX remnants were found embedded in new bone (Figure 3a,b) or integrated into connective tissue (Figure 3c), often infiltrated by fibroblasts and blood vessels, indicating active remodelling.

In two CG1 defects, matrix remnants were associated with localised inflammatory infiltrates, likely due to surgical contamination rather than a material response. Near the root surface, VCMX remnants were sometimes integrated into newly forming bone or the PDL.

Across all groups, regenerated PDL showed normal anatomical architecture (Figure 3d), with regularly organised fibre bundles. In several cases, PDL fibres inserted perpendicularly into newly formed cementum, resembling native periodontal structure.

### 3.3 Histometry

Inter‐rater reliability was excellent (κ = 0.84, 95% CI: 0.80–0.88; *p* < 0.001). Of 48 defects, 4 were excluded because of inflammation (2 CG1 sites) or root damage (1 TG1, 1 CG2), leaving 11 TG1, 12 TG2, 10 CG1 and 11 CG2 defects for histometry. Descriptive statistics and pairwise comparisons are shown in Table 1.

**TABLE 1:** Histometric results.

Group	Height of defect (mm) Mean ± SD	Length of new continuous cementum (mm) mean ± SD	Length of new continuous cementum (%) mean ± SD	Highest point of new cementum (mm) mean ± SD	Highest point of new cementum (%) mean ± SD	Height of new bone (mm) mean ± SD	Height of new bone (%) mean ± SD	Length of JE (mm) mean ± SD	CT Adhesion (mm) mean ± SD
TG1 (VCMX + liquid PRF)	4.67 ± 0.83	4.08 ± 0.88	87.06 ± 8.22	4.42 ± 0.82	94.81 ± 4.01	3.42 ± 0.70	73.29 ± 7.28	1.71 ± 0.31	0.24 ± 0.15
TG2 (Liquid PRF)	4.46 ± 0.40	2.92 ± 1.13	67.92 ± 29.37	4.22 ± 0.26	94.48 ± 5.88	3.19 ± 0.78	69.76 ± 11.45	1.77 ± 0.46	0.37 ± 0.33
CG1 (VCMX)	4.77 ± 0.71	2.07 ± 1.06	46.57 ± 27.54	4.36 ± 0.62	91.67 ± 6.67	3.29 ± 0.77	68.60 ± 7.23	2.21 ± 0.98	0.28 ± 0.34
CG2 (Empty)	4.73 ± 0.59	0.84 ± 0.74	23.98 ± 14.61	3.17 ± 0.41	67.12 ± 6.89	2.58 ± 0.38	54.46 ± 4.77	1.72 ± 0.26	1.35 ± 0.40
*p*‐value TG1 vs. TG2	> 0.999	> 0.999	> 0.999	> 0.999	> 0.999	> 0.999	> 0.999	> 0.999	> 0.999
*p*‐value TG1 vs. CG1	> 0.999	0.265	0.256	> 0.999	> 0.999	> 0.999	> 0.999	> 0.999	> 0.999
*p*‐value TG1 vs. CG2	> 0.999	**0.004**	**0.021**	0.0834	**0.021**	**0.043**	**0.0105**	> 0.999	**0.021**
*p*‐value TG2 vs. CG1	> 0.999	> 0.999	0.705	> 0.999	> 0.999	> 0.999	> 0.999	> 0.999	> 0.999
*p*‐value TG2 vs. CG2	> 0.999	0.084	0.083	**0.043**	**0.043**	0.441	0.083	> 0.999	0.265
*p*‐value CG1 vs. CG2	> 0.999	> 0.999	> 0.999	**0.021**	0.083	0.441	0.083	> 0.999	**0.0237**

Abbreviations: CT, connective tissue; JE, junctional epithelium; mm, millimetres; PRF, platelet‐rich fibrin; SD, standard deviation; VCMX, volume‐stable cross‐linked collagen matrix. Bold values indicate statistical significance (*p* < 0.05).

**FIGURE 4:**
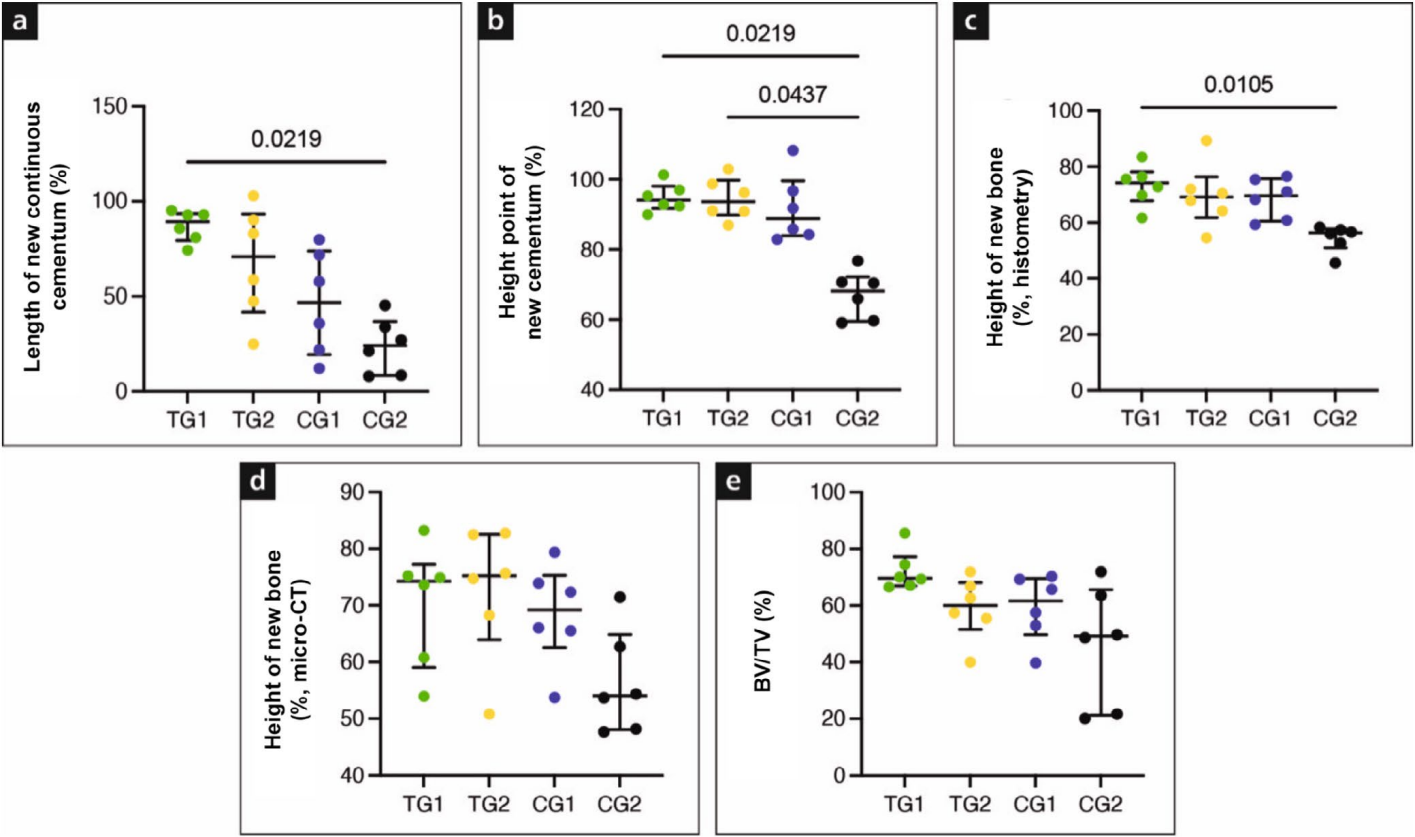
Figure of histometric (a, b, c) and micro‐CT results (d, e). TG1 (VCMX + liquid PRF), TG2 (liquid PRF), CG1 (VCMX) and CG2 (empty, g, h). BV/TV, bone volume/total volume.

Baseline defect heights were comparable across groups (4.46–4.77 mm; *p* > 0.999), confirming consistent defect sizes. TG1 achieved the greatest continuous cementum formation (Figure 4a, 4.08 ± 0.88 mm; 87.1% ± 8.2%), exceeding CG1 (2.07 ± 1.06 mm; 46.6% ± 27.5%; *p* = 0.256) and CG2 (0.84 ± 0.74 mm; 24.0% ± 14.6%; *p* = 0.004, 0.021). TG2 (2.92 ± 1.13 mm; 67.9% ± 29.4%) showed slightly less cementum than TG1 (*p* > 0.999) but tended towards higher values than CG2 (*p* = 0.084, 0.0083).

The highest point of new cementum nearly reached the original defect height in TG1 (Figure 4b, 4.42 ± 0.82 mm; 94.8% ± 4.0%) and TG2 (4.22 ± 0.26 mm; 94.5% ± 5.9%), both significantly higher than CG2 (3.17 ± 0.41 mm; 67.1% ± 6.9%; *p* = 0.021, 0.043).

New bone formation was similar between TG1 (Figure 4c, 3.42 ± 0.70 mm; 72.3% ± 7.3%) and TG2 (3.19 ± 0.78 mm; 69.8% ± 11.5%; *p* > 0.999), both exceeding or trending higher than CG2 (2.58 ± 0.38 mm; 54.5% ± 4.8%; *p* = 0.043, 0.0105 for TG1; *p* = 0.441, 0.083 for TG2). Thus, PRF enhanced bone regeneration regardless of scaffold use.

Length of JE was smaller in TG1 (1.71 ± 0.31 mm) and TG2 (1.77 ± 0.46 mm) without significant group differences (*p* > 0.999). Connective tissue adhesion was minimal in TG1 (0.24 ± 0.15 mm) and TG2 (0.37 ± 0.33 mm), significantly less than CG2 (1.35 ± 0.40 mm; *p* = 0.021 for TG1 vs. CG2).

Overall, TG1 (VCMX + PRF) showed the most favourable outcomes for cementum and bone formation, while TG2 (PRF alone) also improved healing versus CG2. Both PRF‐based approaches outperformed controls, with the combined treatment yielding the most consistent regeneration.

### 3.4 Micro‐CT

**FIGURE 5:**
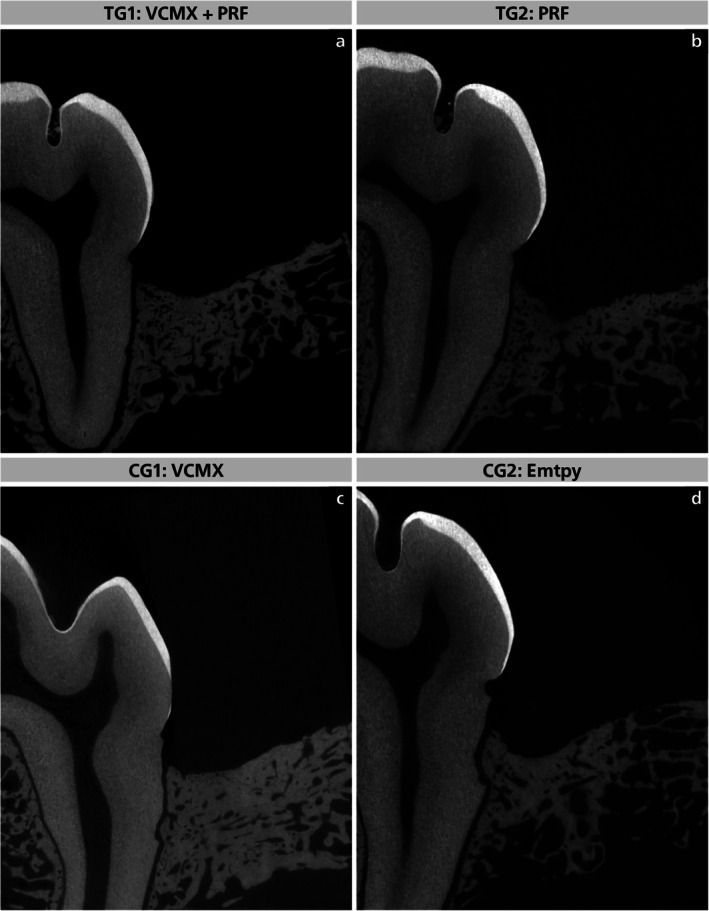
Representative micro‐CT images of TG1 (a), TG2 (b), CG1 (c) and CG2 (d).

The same defects analysed histometrically were also evaluated by micro‐CT, revealing intergroup differences in new bone formation. Detailed values and pairwise comparisons are summarised in Table 2, and representative micro‐CT illustrations are provided in Figure 5.

**TABLE 2:** Results of the micro‐CT analysis.

Group	Height of defect (mm) mean ± SD	Height of new bone (mm) mean ± SD	Height of new bone (%) mean ± SD	BV/TV (%)
TG1	4.70 ± 0.49	3.28 ± 0.62	70.28 ± 10.79	72.21 ± 7.16
TG2	4.53 ± 0.33	3.30 ± 0.69	72.47 ± 11.91	59.05 ± 11.12
CG1	4.68 ± 0.52	3.22 ± 0.68	68.49 ± 8.91	59.29 ± 11.71
CG2	4.57 ± 0.63	2.55 ± 0.41	56.34 ± 9.16	45.94 ± 21.26
*p*‐value TG1 vs. TG2	> 0.999	> 0.999	> 0.999	> 0.999
*p*‐value TG1 vs. CG1	> 0.999	> 0.999	> 0.999	0.705
*p*‐value TG1 vs. CG2	> 0.9999	0.705	0.265	0.441
*p*‐value TG2 vs. CG1	> 0.999	> 0.999	> 0.999	> 0.999
*p*‐value TG2 vs. CG2	> 0.999	0.152	0.152	> 0.999
*p*‐value CG1 vs. CG2	> 0.999	0.705	> 0.999	> 0.999

The mean defect height was consistent across groups (4.53–4.70 mm; *p* > 0.999). TG2 showed the largest new bone height (Figure 4d, 3.30 ± 0.69 mm; 72.47% ± 11.91%), followed by TG1 (3.28 ± 0.62 mm; 70.28% ± 10.79%), CG1 (3.22 ± 0.68 mm; 68.49% ± 8.91%) and CG2 (2.55 ± 0.41 mm; 56.34% ± 9.16%). Thus, both PRF‐treated groups and CG1 achieved greater bone regeneration than CG2, with TG2 showing the highest values overall.

Bone volume fraction (BV/TV, Figure 4e) was highest in TG1 (72.21% ± 7.16%), followed by CG1 (59.29% ± 11.71%), TG2 (59.05% ± 11.12%) and CG2 (45.94% ± 21.26%). TG1 showed higher new bone height and BV/TV than CG2, although not statistically significant. TG2 also had a higher new bone height percentage than CG2 (*p* = 0.265). No significant differences were found between TG1 and TG2 or between TG1 and CG1 (*p* > 0.05). These findings indicate that PRF enhances bone regeneration compared with the negative control, while the combination with VCMX yields the highest—but not statistically superior—outcomes compared with PRF alone or VCMX alone.

## 4 Discussion

This preclinical study assessed the regenerative potential of liquid PRF, alone or combined with VCMX, in acute, one‐wall intrabony defects. Both treatments significantly enhanced periodontal regeneration, with the VCMX + PRF group (TG1) showing the best outcomes in continuous cementum and new bone formation. TG1 achieved significantly greater continuous cementum formation than both controls, while PRF alone (TG2) also outperformed the empty control (CG2). Micro‐CT confirmed these results, showing higher bone volume fraction and new bone height in both PRF‐treated groups, particularly TG1. These findings highlight the strong regenerative potential of liquid PRF—especially when combined with a scaffold—for promoting early periodontal regeneration in non‐contained defects.

The enhanced regenerative outcomes observed with VCMX and PRF likely result from a combination of mechanical and biological effects. The VCMX scaffold provided space maintenance and blood clot stabilisation, both critical factors for periodontal regeneration (Sculean et al. 2015; Susin et al. 2015). Simultaneously, PRF, rich in platelets, leukocytes and growth factors, likely stimulated angiogenesis, cellular migration and proliferation, thereby accelerating tissue maturation (Choukroun and Ghanaati 2018; Dohle et al. 2018; Fujioka‐Kobayashi et al. 2017; Ma et al. 2023). Importantly, PRF's biological effects extend beyond simple wound healing to modulation of inflammation. Several studies have demonstrated that PRF reduces the production of pro‐inflammatory cytokines, promotes macrophage polarisation towards an M2 regenerative phenotype and inhibits key inflammatory mediators such as IL‐1β and TNF‐α (Kargarpour et al. 2021a, 2021b; Kargarpour et al. 2023; Nasirzade et al. 2020; Sordi et al. 2022). Such immunomodulatory effects are pivotal for creating an anti‐inflammatory and pro‐regenerative micro‐environment.

Recent systematic reviews have confirmed that autologous platelet concentrates, particularly PRF, substantially enhance periodontal regenerative outcomes compared to open flap debridement alone or in combination with bone grafts (Miron, Moraschini, et al. 2025a). Notably, PRF has been shown to improve probing depth reduction, clinical attachment level gain and radiographic bone fill, reinforcing its versatile regenerative potential (Miron, Moraschini, et al. 2025a). Emerging studies have further emphasised the benefits of liquid PRF, which, owing to its superior growth factor release and regenerative properties, can significantly enhance periodontal wound healing (Miron et al. 2023). Liquid PRF has also demonstrated the ability to promote fibroblast migration, stimulate angiogenesis and exhibit antimicrobial effects against periodontal pathogens (Blatt et al. 2021).

Histological analysis in the present study revealed that TG1 defects exhibited more pronounced VCMX degradation and integration into newly formed tissues compared to CG1, suggesting a synergistic effect between PRF and the collagen matrix. However, the cementum outcomes indicate that the biological effect was primarily attributable to PRF, as both TG1 and TG2 performed better than the empty control while differences among TG1, TG2 and CG1 were mostly statistically non‐significant. This observation aligns with the findings by Ratajczak et al. (2018), who showed that the fibrin matrix in PRF traps and gradually releases bioactive molecules, thus enhancing vascularisation and tissue maturation. Furthermore, Bartold and Ivanovski (2025) emphasised that autologous platelet concentrates support wound healing by modulating inflammation, promoting angiogenesis and remodelling the extracellular matrix.

Liquid PRF alone also demonstrated significant regenerative benefits, notably improving continuous cementum formation and positioning the highest point of new cementum more coronally compared to CG2. However, outcomes with liquid PRF alone were slightly inferior to those observed with the combined approach, highlighting the critical role of scaffold‐based space maintenance, particularly in non‐contained defects. In addition to providing mechanical stability, the collagen matrix may also serve as a carrier and controlled release system for growth factors contained in PRF (Al‐Maawi et al. 2019). This dual function could contribute to the more consistent and predictable regenerative outcomes observed in TG1. In contrast, the absence of a scaffold in TG2 may have resulted in more variability in regenerative patterns. This is also reflected by the wider distribution of histometric values, as shown in Table 1. These findings are consistent with clinical studies reporting enhanced healing outcomes when PRF is incorporated into scaffold‐supported regenerative procedures (Dohle et al. 2018; Fujioka‐Kobayashi et al. 2017).

Both TG1 and TG2 exhibited significantly greater new bone formation compared to CG2. However, no significant differences were detected between TG1, TG2 and CG1 regarding bone regeneration. This suggests that while PRF markedly enhances soft‐tissue and cementum regeneration, its osteogenic potential may depend on additional structural support, such as that provided by collagen scaffolds. Supporting this, meta‐analyses have shown that the combination of PRF with bone grafts achieves superior outcomes compared to bone grafts alone (Miron, Moraschini, et al. 2025a; Silva et al. 2024).

The present study has several strengths, including the use of a robust and well‐established preclinical model. Nevertheless, some limitations should be acknowledged. From an ethical perspective, the use of a canine model warrants consideration within the framework of the 3Rs (Replacement, Reduction, Refinement). The dog model was selected because it represents one of the few translationally relevant systems that closely resembles human periodontal wound healing, thereby providing information not achievable with small‐animal or in vitro models (Kantarci et al. 2015). Reduction was achieved by adopting a split‐mouth design that minimised the number of animals while allowing multiple experimental sites per subject. Refinement was ensured through standardised surgical procedures, advanced perioperative care and close daily monitoring, which collectively minimised animal suffering and optimised welfare. In addition, the use of surgically created acute defects may not fully replicate the chronic inflammatory environment of periodontitis. The 12‐week observation period was selected as it represents a standard time point in preclinical periodontal regeneration studies, allowing assessment of early to mid‐term healing and comparison with previous investigations (Bommer et al. 2022; Kantarci et al. 2015; Nyman et al. 1980). However, it does not provide information on the long‐term stability of regenerated tissues, which should be addressed in future clinical studies. Finally, no biological characterisation of the PRF preparations was performed, which precludes conclusions about their cellular and molecular composition. Consequently, future studies are warranted to investigate early wound healing dynamics, scaffold degradation profiles and the short‐ and long‐term clinical performance of PRF‐based therapies, particularly in chronic defect models and human clinical trials.

## 5 Conclusion

In conclusion, the present findings indicate that (i) the use of liquid PRF alone or in combination with VCMX enhances periodontal regeneration to a greater extent than open flap debridement alone, and (ii) the combination tended to show the most favourable outcomes. These differences were mostly not statistically significant compared to PRF alone or VCMX alone, suggesting that the main regenerative effect was driven by PRF, with the scaffold providing additional structural support.

## Author Contributions

J.‐C.I., A.St. and A.S. conceived the idea; J.‐C.I., A.R. and M.P.M. performed the clinical procedures; J.‐C.I., A.R., S.C.I. and M.P.M. collected the data; J.‐C.I., D.D.B. and A.St. analysed the data; J.‐C.I. and A.R. led the writing. All authors read and approved the final version of the manuscript.

## Funding

This study was funded by a grant from the Osteology Foundation (Grant number 19–147).

## Ethics Statement

The current study was conducted in accordance with the European Communities Council Directive 2010/63/EU, approved by the Ethics Committee of the Rof Codina Foundation, Lugo, Spain (03/20/LU‐001).

## Conflicts of Interest

This study was funded by a grant from the Osteology Foundation. Both Jean‐Claude Imber and Alexandra Stähli receive grants from the Geistlich‐Stucki Foundation, Switzerland. All authors report having no other potential conflicts of interest related to this study.

## Data Availability

The data that support the findings of this study are available from the corresponding author upon reasonable request.
